# Extraction of Antioxidant Compounds from Onion Bulb (*Allium cepa* L.) Using Individual and Simultaneous Microwave-Assisted Extraction Methods

**DOI:** 10.3390/antiox11050846

**Published:** 2022-04-26

**Authors:** Ana V. González-de-Peredo, Mercedes Vázquez-Espinosa, Estrella Espada-Bellido, Marta Ferreiro-González, Ceferino Carrera, Gerardo F. Barbero, Miguel Palma

**Affiliations:** Department of Analytical Chemistry, Faculty of Sciences, Agrifood Campus of International Excellence (ceiA3), IVAGRO, University of Cadiz, 11510 Cadiz, Spain; ana.velascogope@uca.es (A.V.G.-d.-P.); mercedes.vazquez@uca.es (M.V.-E.); estrella.espada@uca.es (E.E.-B.); marta.ferreiro@uca.es (M.F.-G.); ceferino.carrera@uca.es (C.C.); miguel.palma@uca.es (M.P.)

**Keywords:** *Allium cepa* L., anthocyanins, Box-Behnken, HPLC, microwave-assisted extraction, onion, phenolic compounds

## Abstract

Despite the excellent beneficial properties that anthocyanins and total phenolic compounds give to the red onion bulbs, few articles have investigated modern extraction techniques or experimental designs in this field. For this reason, the present study proposes the development and optimization of alternative methods for the extraction of these compounds based on microwave-assisted extraction and the Box-Behnken experiment design. The optimal values for the extraction of total anthocyanins have been established at 62% methanol composition as a solvent, pH 2, 56 °C temperature, and 0.2:13 g:mL sample-solvent ratio. Regarding the extraction of total phenolic compounds, the optimal conditions have been established at 100% pure methanol as a solvent with pH 2, 57 °C temperature, and 0.2:8.8 g:mL sample-solvent ratio. Short extraction times (min), good recoveries (mg of bioactive compound g^−1^ of dry onion), and high repeatability and intermediate precision (coefficient of variation (%)) have been confirmed for both methods. Regarding total anthocyanins, the following results have been obtained: 2 min, 2.64 ± 0.093 mg of total anthocyanins g^−1^ of dry onion, and 2.51% and 3.12% for precision. Regarding phenolic compounds, the following results have been obtained: 15 min, 7.95 ± 0.084 mg of total phenolic compound g^−1^ of dry onion, and 3.62% and 4.56% for precision. Comparing these results with those of other authors and with those obtained in a previous study of ultrasound-assisted extraction, it can be confirmed that microwave-assisted extraction is a quantitative, repeatable, and very promising method for the extraction of phenolic compounds and anthocyanins, which offers similar and even superior results with little solvent expense, time, and costs.

## 1. Introduction

Phenolic compounds include a wide range of compounds with an important capacity to avoid the oxidation of free radicals and, therefore, to avoid some of the various diseases that are caused by these radicals [1,2]. Anthocyanins are a specific type of phenolic compound that is broadly found in nature [3]. They are quite easy to identify thanks to the red, purple, or blue color that they confer to certain parts of plants (leaves, fruits, flowers, etc.) [4]. From the chemical point of view, they generally appear as glycosides or acylglycosides of anthocyanidins, the aglycones, and vary according to different hydroxyl or methoxyl substitutions of their basic flavylium (2-phenylbenzopyrilium) structure [5]. These compounds have multiple functions in plants, as they may act as a protective substance against UV radiation or as a luring element for pollinating insects [6]. With regard to human health, the beneficial effect of anthocyanins as natural antioxidants has attracted considerable interest [7]. Anthocyanins are efficacious, for example, as natural antioxidants, anti-inflammatory, anticancer, anti-Alzheimer, or anti-Parkinson [8].

Although anthocyanins have always been found in fruits and berries in large amounts [9,10], they can also be found in certain vegetables. In fact, some vegetables have proven to have a high content of conjugated anthocyanins, which are believed to be of greater interest to the food industry because of their increased stability [11]. Red onion, which owns its color mainly to the anthocyanins that can be found in the epidermal cells of the bulb’s scale leaves, is one of the vegetables that stands out for its anthocyanins content [12]. Specifically, the most common anthocyanins that are present in red onions are cyanidin derivatives, with a superior content of cyanidin 3-*O*-(6″-malonylglucoside), although peonidin and delphinidin derivatives have also been identified to a lesser extent [13,14,15].

Even though many articles have studied the anthocyanins in onion for their beneficial properties regarding consumers’ health, they seem to have exclusively focused on their identification and analysis, while not enough attention has been paid to the amount of bioactive compounds extracted [16,17,18]. After revising the bibliography on the subject from 1994 until now, most of the studies have employed traditional techniques such as maceration or stirring for extraction purposes [19,20,21,22]. The use of more modern, more efficient, greener, and faster techniques has hardly been exploited even at the laboratory level. Techniques such as ultrasound-assisted extraction (UAE) or microwave-assisted extraction (MAE) are excellent alternatives that can achieve better results in shorter times and, consequently, with lesser solvent consumption and lower costs [23,24]. In a previous study published by our research group [25], the main anthocyanins and the total phenolic compounds present in red onion were determined by employing UAE. In the present study, however, MAE is presented as the alternative to more traditional extraction methods since this technique may achieve extractions that are comparable or even greater than those obtained by UAE.

MAE is a novel extraction technique based on the use of microwave energy to extract substances that are soluble in a fluid. Microwaves energy is generated by electromagnetic fields within a frequency range that goes from 300 MHz to 300 GHz [26]. This energy penetrates materials and causes temperature increments through polar components, rotation of dipoles, and the conductive migration of dissolved ions. This localized rise in temperature generates, in turn, a rise of pressure that can induce the selective migration at a rapid rate of the target compounds from the material to the extraction solvent [27]. So, MAE presents some advantages, such as shorter extraction times and lesser solvent consumption, on top of the possibility of simultaneously extracting multiple samples [28].

In the revision of the bibliography on this subject, one study where MAE was used as the extraction technique to obtain anthocyanins from onions has been found just [29]. This article investigated how the concentration of the solvents, the acids, the different temperatures, and an array of extraction times had an influence on the extraction of the anthocyanins. However, an experimental design methodology was not used, and the variables were studied individually (one factor at a time) instead. For that reason, this study was intended to highlight the importance of employing a Design of Experiments (DOE) together with a Response Surface Methodology (RSM) to optimize the variables involved in the extraction of the compounds of interest [30]. In this case, the extractions of the anthocyanins and the total phenolic compounds present in onion matrices have been evaluated. The results from the DOE are subjected to RSM to generate the mathematical model that best fits the data obtained and to establish the optimal values of the factors that have an influence on the response variable [31]. For this study, a three-level fractional factorial Box-Behnken design has been selected, and the most relevant variables in MAE have been established as follows: methanol percentage in the solvent, solvent pH, sample:solvent volume ratio, and temperature.

Therefore, the present study intends to develop and optimize two Microwave-Assisted Extraction (MAE) methods to obtain anthocyanins and total phenolic compounds from red onions. The importance of the combined use of an experimental design with such a microwave-assisted extraction technique for optimization purposes has been particularly emphasized. By combining these modern techniques, more efficient and productive extractions should be obtained so that larger amounts and more quality products are offered to consumers.

## 2. Materials and Methods

### 2.1. Chemicals and Standards

In the present study, different chemicals for the MAE as well as for the quantification of anthocyanins, total phenolic compounds, and antioxidant activity have been employed as follows: methanol of HPLC purity (Fischer Chemical, Loughborough, UK), Milli-Q water from a Milli-Q water purification system (Millipore, Bedford, MA, USA), hydrochloric acid (Panreac, Barcelona, Spain), sodium hydroxide (Panreac, Barcelona, Spain), formic acid (Scharlau, Barcelona, Spain), anhydrous sodium carbonate (Panreac Química, Castellar del Valles, Barcelona, Spain), Folin–Ciocalteu reagent (Merck KGaA, EMD Millipore Corporation, Darmstadt, Germany), and 2,2-diphenyl-1-picrylhydrazyl (DPPH) (Sigma-Aldrich, San Luis, MO, USA). Furthermore, for the quantification of anthocyanins, total phenolic compounds, and antioxidant activity, three standards were employed respectively: cyanidin chloride, gallic acid, and 6-hydroxy-2,5,7,8-tetramethylchroman-2-carboxylic acid (Trolox), all of them supplied by Sigma-Aldrich Chemical Co. (St. Louis, MO, USA).

### 2.2. Biological Material Preparation

Homogeneous lyophilized red onion was the biological material used for the optimization of the extraction methods. The characteristics of the onions were the following: red color, caliber 50/90 mm, origin Austria (Cebollas Tara, S.L., Requena, Spain). After purchasing red onions from a local market in the province of Cadiz (Spain), they were subjected to a specific pretreatment. The outer layers of the onion bulbs were removed, and their cores were cut into small pieces. Then, they were lyophilized by means of an LYOALFA freeze dryer (Azbil Telstar Technologies, Terrasa, Spain) and crushed employing a ZM200 knife mill (fineness <300 μm, Retsch GmbH, Haan, Germany). The homogeneous lyophilized red onion samples were kept in a freezer (−20 °C) prior to analysis.

### 2.3. Microwave-Assisted Extraction Procedure

The extraction equipment used was a MARS 6 One TouchTM Technology system (1800 W) (CEM Corporation, Matthews, NC, USA), specifically designed for routine sample preparation. For the extraction processes, approximately 0.2 g of homogenized red onion samples were weighed in 75 mL MARSXpress vessels (CEM Corporation), and the corresponding volume of solvent (formed by different proportions of methanol:water mixtures with varying pH) was added according to the experimental design. Nine vessels containing the same solvent and volume were securely closed and placed into the microwave oven. As mentioned above, the solvent volume was selected according to the experiment to be carried out; similarly, the rest of the factors to be considered (temperature, percentage of methanol in the solvent, and pH) were set according to the corresponding experimental conditions as follows: 50% methanol in water to 100% pure methanol as solvent, 2 to 7 pH, 50 to 100 °C temperature, and 0.2:10 to 0.2:20 g:mL as the sample:solvent ratio. Five minutes was set as the initial time (followed by the subsequent cooling time) for MARS application. The liquid obtained from each extraction was transferred to centrifuge tubes and centrifuged at 1702 g for 5 min. The supernatant was added to a 25 mL volumetric flask, and the precipitates were subsequently redissolved in 5 mL of the same extraction solvent and centrifuged again. The second supernatant was poured into the same volumetric flasks and made up to the mark using the same solvent. The extracts were stored in a freezer (−20 °C) prior to analysis.

### 2.4. Total-Phenolic Compounds

The Total Phenolic Compounds (TPC) in the red onion extracts were determined by Folin–Ciocalteu assay as described by Singleton and Rossi with some modifications [32]. The calibration curve was constructed using gallic acid at different concentrations: 0.5, 1.0, 5.0, 10.0, 25.0, and 50.0 mg L^−1^ in distilled water. Water (1.25 mL), Folin–Ciocalteu reagent (12.5 mL), and an aqueous solution of sodium carbonate at 20% p/v (5 mL) were added to 0.25 mL of each gallic acid concentration and, after 30 min at room temperature, the absorbances of the standard curve were read at 765 nm by means of a Cary 4000 UV-Vis (Agilent, Santa Clara, CA, USA). The plot of absorbance vs. concentration using Microsoft Office Excel 2013 resulted in the following regression equation (y = 0.0014x + 0.0022) and linear relationship (R^2^ = 0.9995). Then, the same method was applied to the MAE samples after filtering the extracts through a 0.45 µm nylon filter (Membrane Solutions, Dallas, TX, USA) before spectrophotometric analysis. Finally, their absorbances were expressed as mg of gallic acid equivalents per gram of dried red onion (mg GAE g^−1^) according to the calibration curve.

### 2.5. Antioxidant Activity

The antioxidant activity of the red onion extracts was determined by employing the DPPH assay and following the method described by Miliauskas et al. with some modifications [33]. The DPPH assay is one of the most used when studying antioxidant capacity [34] since it also has short analysis times as an advantage [35,36]. The calibration curve was constructed using a Trolox standard at different concentrations: 0.0, 0.3, 0.6, 0.9, 1.1, and 4.4 mmol L^−1^ in methanol. 2 mL of the DPPH solution (6 × 10^−5^ mol L^−1^ prepared in methanol) were added to 100 µL of each Trolox concentration and, after 40 min at room temperature and in the absence of light, the absorbances of the standards were read at 515 nm using a Cary 4000 UV-Vis (Agilent, Santa Clara, CA, USA). The absorbance vs. concentration curve was represented using Microsoft Office Excel 2013, which resulted in the following regression equation (y = 883941x + 0.7478) and linear relationship (R^2^ = 0.9959). Then, the same method was applied to the MAE samples after filtering the extracts through a 0.45 µm nylon filter (Membrane Solutions, Dallas, TX, USA) before spectrophotometric analysis. Finally, the absorbance data were expressed as mg of Trolox equivalents (TE) per gram of dried red onion (mg TE g^−1^ dry onion) according to the calibration curve.

### 2.6. UHPLC-MS-QToF Conditions

The anthocyanins extracted from the red onion samples were identified using Ultra-High-Performance Liquid Chromatography (UHPLC) coupled to a quadrupole time-of-flight mass spectrometer (Q-ToF-MS) (Xevo G2 QToF, Waters Corp., Milford, MA, USA). Specifically, the identification was carried out following the method previously published by our research group [17]. By applying this method, 9 different anthocyanins were identified based on their retention time and molecular weight: cyanidin 3-*O*-glucoside (3.517 min, *m/z* 449.1087), cyanidin 3-*O*-laminaribioside (4.132 min, *m/z* 611.1641), cyanidin 3-*O*-(3″-malonylglucoside) (4.875 min, *m/z* 535.1069), peonidin 3-*O*-glucoside (5.384 min, *m/z* 463.1251), delphinidin 3,5-*O*-diglucoside (5.721 min, *m/z* 649.1392), cyanidin 3-*O*-(6″-malonylglucoside) (5.850 min, *m/z* 535.1104), cyanidin 3-*O*-(6”-malonyl-laminaribioside) (6.052 min, *m/z* 697.1613), and peonidin 3-*O*-(6″-malonylglucoside) (6.323 min, *m/z* 549.1255), and delphinidin 3-*O*-glucoside (6.536 min, *m/z* 487.0863).

### 2.7. HPLC Conditions

**Figure 1:**
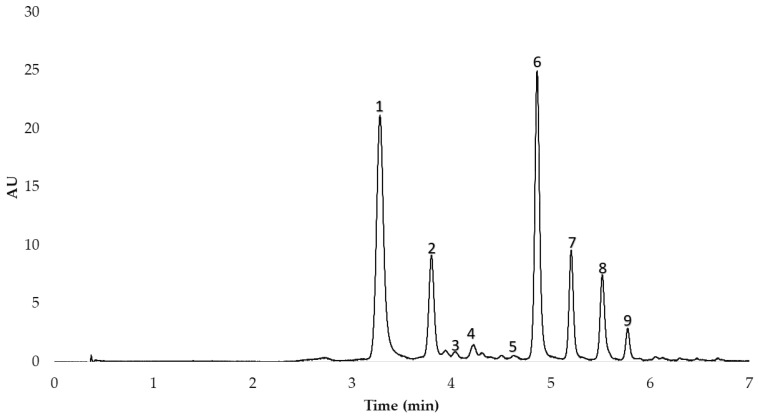
HPLC chromatogram of the nine anthocyanins identified in the MAE extracts from red onion. 1. cyanidin 3-*O*-glucoside; 2. cyanidin 3-*O*-laminaribioside; 3. cyanidin 3-*O*-(3″-malonylglucoside); 4. peonidin 3-*O*-glucoside; 5. delphinidin 3,5-*O*-diglucoside; 6. cyanidin 3-*O*-(6″-malonylglucoside); 7. cyanidin 3-*O*-(6″-malonyl-laminaribioside); 8. peonidin 3-*O*-(6″-malonylglucoside); 9. delphinidin 3-*O*-glucoside.

After the anthocyanins in the red onion extracts had been identified, they were separated and quantified by means of High-Performance Liquid Chromatography (HPLC) with the equipment available in our research group (Elite HPLC LaChrom Ultra System, Hitachi, Tokyo, Japan) coupled to an L-2420U UV-Vis detector, an L-2200U autosampler, an L-2300 column oven, and two L-2160 U pumps. Specifically, the analysis was carried out following the method previously published by our research group [17]. The column employed was a reverse-phase C18 analytical column (2.6 µm, 2.1 mm × 100 mm, Phenomenex, Torrance, CA, USA). The gradient allowed the optimum separation of the 9 peaks in less than 6 min. The HPLC chromatogram obtained is shown in Figure 1.

Cyanidin chloride was used as the standard for quantification purposes. A plot of area vs. concentration (0.06–35 mg L^−1^) was generated by means of Microsoft Office Excel 2013, and the following regression equation (y = 260596.88x − 4292.66) and linear relationship (R^2^ = 0.9999) were obtained. A calibration curve was plotted for each one of the remaining anthocyanins detected on the basis that all the anthocyanins have similar absorbance and by taking into account their molecular weights. So, the area corresponding to each anthocyanin as quantified by HPLC was expressed as mg of the corresponding anthocyanin per gram of dried red onion (mg g^−1^) according to the calibration curve.

### 2.8. Optimization Procedure and Data Analysis

A Box-Behnken design (BBD) together with RSM was used for the optimization of several experimental parameters involved in the extraction of bioactive compounds from red onion. BBD is a class of rotatable or nearly rotatable second-order designs based on three-level incomplete factorial designs [37]. In this work, the effect of 4 independent variables, namely the percentage of methanol in the solvent, the solvent pH, the extraction temperature, and the ratio between sample mass and solvent volume on the response (Total Phenolic Compounds (TPC), and Total Anthocyanins (TA)) have been investigated. As these independent variables have different units and ranges, they were normalized and coded between −1 and +1 (three levels) to obtain a more uniform response. The range of the independent variables and their levels were the following: 50(−1)-75(0)-100(1) percentage of methanol in the solvent (%); 2(−1)-4.5(0)-7(1) solvent pH, 50(−1)-75(0)-100(1) extraction temperature (ºC); and 0.2:10(−1)-0.2:15(0)-0.2:20(1) ratio between the sample mass and solvent volume (g:mL). The number of experiments (N) required was determined by applying the following BBD equation (Equation (1)):

*N* = 2*k*(*k* − 1) + *C*_0_, (1)

where *k* is the number of factors and *C*_0_ is the number of central points. In this work, with 4 factors and 3 central points, a BBD design comprising 27 experimental points was carried out at random. These experiments and their results can be seen in Table 1.

Based on the BBD results, the RSM can be employed to produce a regression model for each response. This mathematical model can be used to generate a predictive equation (Equation (2)) for each response (total phenolic compounds and total anthocyanins) based on the experimental conditions employed. Furthermore, an analysis of variance (ANOVA) was conducted to verify by means of the application Statgraphics Centurion version XVI (Warrenton, VA, USA) the suitability of the model obtained.

(2) $$Y={\;\beta }_{0}+\overset{k}{\underset{i=1}{\sum }}\beta_{i}X_{i}+{\;\beta }_{\mathrm{ii}\;}X_{i}^{2}+\underset{i}{\sum }\overset{k}{\underset{i=1}{\sum }}\beta_{\mathrm{ij}}X_{i}X_{j}+r$$

where Y represents the responses; β_0_ the model constant; β_i_ the coefficient for each main effect; β_ij_ the coefficient corresponding to the interactions between factor i and factor j; β_ii_ the coefficient of the quadratic factors that represent the curvature of the surface; X each one of the factors studied; and r the residual value (random error).

Once the individual response surfaces had been determined for each response, a multi-response optimization for both responses was performed following the desirability function. The predicted values obtained from each response surface were transformed into a dimensionless scale *d_i_*. The geometric means of each individual desirability value were combined to obtain the overall desirability *D*, and an algorithm was applied to the *D* function to determine the set of variable values that maximize it [38]. All of these calculations were also carried out by means of the application Statgraphics Centurion version XVI (Warrenton, VA, USA). The *t*-test was used to compare the results obtained from the independent and from the multi-response methods.

## 3. Results and Discussion

### 3.1. Temperature Stability Study

**Figure 2:**
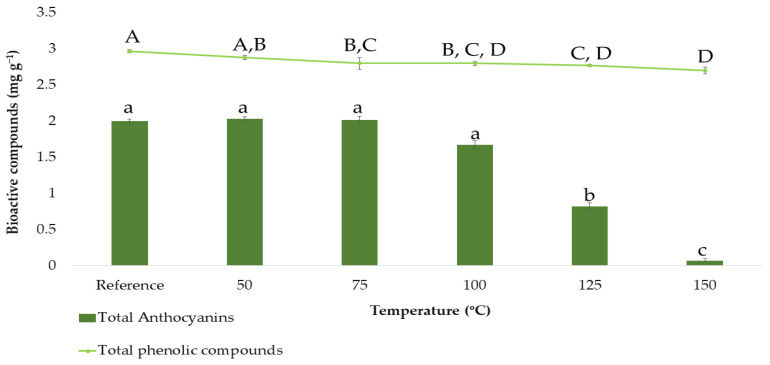
Stability study on total anthocyanins and total phenolic compounds content (means ± SD). Different letters mean statistically significant differences according to Tukey’s test at the 95% level of significance.

In order to determine the total phenolic compounds and the total anthocyanins based on the BBD design, the ranges corresponding to each factor had to be previously established. Except for that corresponding to the temperature, the rest of the ranges were set according to the group’s previous experience with onion matrices [25]. Since temperatures are higher in microwave-assisted extraction processes than in ultrasound-assisted extraction ones, this variable was studied separately by carrying out a stability study of the extracted bioactive compounds. For that purpose, the following extraction temperatures were studied in triplicate: 50, 75, 100, 125, and 150 °C, while the rest of the factors were set at intermediate constant values (50% methanol:water, 0.2:15 g:mL ratio and 5 min extraction time). The results obtained can be seen in Figure 2.

Figure 2 shows how the anthocyanins content varies as a function of the temperature. The reference sample and the samples subjected to 50, 75, and 100 °C showed similar total anthocyanins contents according to Tukey’s test (group a). Above 100 °C, a clear decrease in total anthocyanins content was observed, which fell as low as 1 mg g^−1^ or less when the temperature reached its maximum level at 150 °C. This reduction is clearly due to the degradation experienced by the anthocyanins, as these compounds are characterized by their thermolability, and they had been exposed for 5 min to microwave extractions at high temperatures [39,40]. Consequently, 50–75–100 °C were the values to be incorporated into the BBD. On the other hand, Figure 2 also shows how the content of total phenolic compounds varies as a function of the temperature, even if these compounds were not so drastically degraded as can be concluded from Tukey’s test. This is so because total phenolic compounds include, in addition to anthocyanins, other compounds such as flavanols, which are less thermolabile. Even though the degradation of total phenolic compounds was not that notable, 50–75–100 °C were also selected as the temperature levels to be incorporated into the BBD since the amount of total phenolic compounds extracted at higher temperatures was also lower.

### 3.2. Box-Behken Designs and RSM

Once the ranges to be considered for each one of the experimental factors had been established, RSM and ANOVA were applied to the experimental matrix of the BBD (Table 1). The data corresponding to both response variables are shown in Table 2 and Table 3. Based on the results obtained, it could be confirmed that both analyses explain 95.28% (for total anthocyanins) and 78.12% (for total phenolic compounds) of the total variability. Furthermore, no significant lack of fit (0.38 for total anthocyanins, and 3.99 for total phenolic compounds) was revealed in either model, which corroborates their good fit.

**Figure 3:**
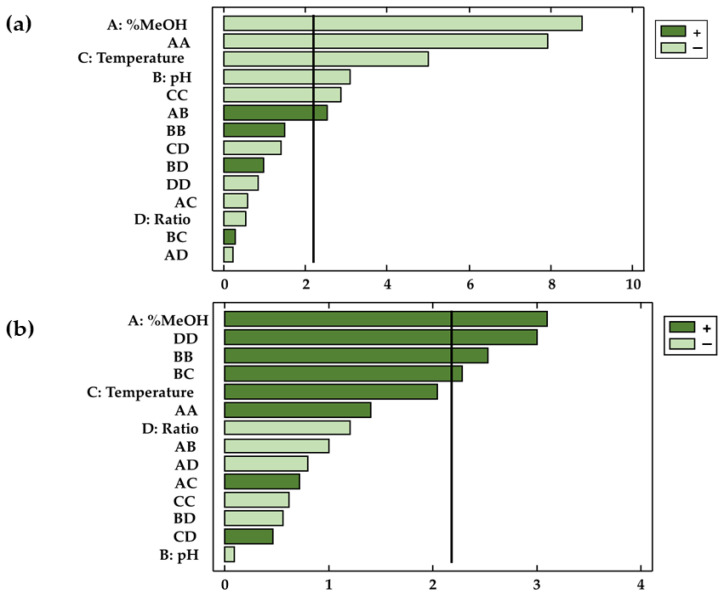
Pareto diagrams for the two responses: (**a**) total anthocyanins and (**b**) total phenolic compounds.

Not only the validity of the models has been confirmed, but this study has also determined the coefficients of the different parameters in the polynomial equation as well as their significance on the dependent variable. The factors and/or interactions that showed *p*-values < 0.05 were considered relevant (with statistical significance) for the response at a given significance level of 95%. This information was complemented by Pareto diagrams (Figure 3a,b), where the influence of the factors or their interactions can be conveniently visualized.

The results obtained showed that the characteristics of the solvent (X_1_), the temperature (X_3_), and the pH (X_2_) had a significant effect on the extracted amounts of anthocyanins *(p*-values < 0.05). On the other hand, with regard to phenolic compounds, only methanol (X_1_) represented a significant factor with respect to the extractions obtained. The results agree with those found in the bibliography, according to which the relation between the percentages of methanol and water in the solvent is a highly influential variable regarding the extraction of phenolic compounds and anthocyanins from natural matrices [41,42,43]. In the case of the bioactive compounds studied in this work, methanol had opposite effects on the extraction of anthocyanins and total phenolic compounds. Thus, while in the case of anthocyanins extraction, methanol had a negative effect on the response variable (b_1_ = −0.51), higher percentages of methanol produced larger extractions of phenolic compounds (b_1_ = 0.63). In the case of the solvent range studied, it was concluded that the extraction of anthocyanins was favored by solvents formed by similar percentages of methanol and water. In regards to total phenolic compounds, larger amounts were obtained when the percentage of methanol in the solvent was higher than that of water. Due to the importance of the similar polarity between the bioactive compounds to be extracted and the solvent, it can be concluded that in addition to anthocyanins, other bioactive compounds are extracted (flavonoids and phenolic acids, among others) when total phenolic compounds are studied. Such compounds, which are also present in onion matrices, are less polar than anthocyanins and are therefore better extracted when using less polar solvents (with more content of methanol than water). The extraction is favored with this similarity of polarities, which intensifies the molecular forces and improves the solubility of the targeted compounds in the solvent [44,45]. With respect to anthocyanins, their solubility depends largely on their chemical structure. Anthocyanins are glycosylated derivatives of anthocyanidins [46], so on the one hand, the flavylium cation is responsible for the water solubility of these compounds [47], while on the other hand, the polyphenolic structure of the anthocyanin adds a hydrophobic characteristic, and makes them soluble in organic solvents, such as ethanol and methanol [48]. Specifically, among the anthocyanins present in onions, delphinidin has the highest polarity, followed by cyanidin [49]. Peonidin has the lowest polarity, probably due to the presence of a methoxy group in the 3′ position of ring B [50]. All these characteristics make the studied anthocyanins soluble both in water and in organic solvents such as methanol in this case. Specifically, a binary mixture of both would be the optimal extraction solvent because it would favor the diffusion of all the anthocyanins from the onion matrix to the solvent during extraction. Furthermore, it is important to note that in MAE, methanol improves the relative permittivity (the dielectric constant) [50], which in turn can improve the rate of internal diffusion and help the solvent to penetrate the solid phase. With respect to total phenolic compounds, due to the mixture of compounds extracted, with more complex and disparate structures and therefore with greater resistance to mass transfer, pure solvents such as methanol offer better results [51,52].

Apart from the extraction solvent, pH also exhibited an inverse relationship with the amount of anthocyanins extracted (b_2_ = −0.17). According to the bibliography, the extraction of anthocyanins is favored at acid pH because they play an important role in the rupture of cell membranes by acid hydrolysis, releasing the anthocyanins bound in the onion matrix and thus enhancing mass transfer [53,54,55]. It should also be kept in mind that the chemical structure of anthocyanins is influenced by the pH range, reaching unstable structures at basic pH. Specifically, anthocyanins are more stable in media with a pH between 1 and 3 [53,56] with the formation of flavylium cation with a red color [57].

Furthermore, temperature has also been reported as an influential extraction factor because of the previously mentioned thermolability of these compounds. Thus, since the temperature has an inverse effect on the response (b_3_ = −0.25), an increase in the extraction temperature has a negative effect on the amounts obtained of the said compounds in the extracts. Although, in many cases, high temperatures facilitate the extraction of the target compounds by softening the tissues, increasing the solubility and the diffusion coefficient [58], in the case of anthocyanins, due to their thermolabile nature, the effect of temperature on the extraction is negative [59]. Temperature can cause degradation of anthocyanins either by hydrolysis of the glycoside, generating unstable forms of aglycone, or by the hydrolytic opening of the heterocyclic ring [60]. Furthermore, if the medium is acidic, as has just been reported, high temperatures promote the hydrolysis of acid components and sugar residues, inactivating the chemical structure of anthocyanins [61].

**Figure 4:**
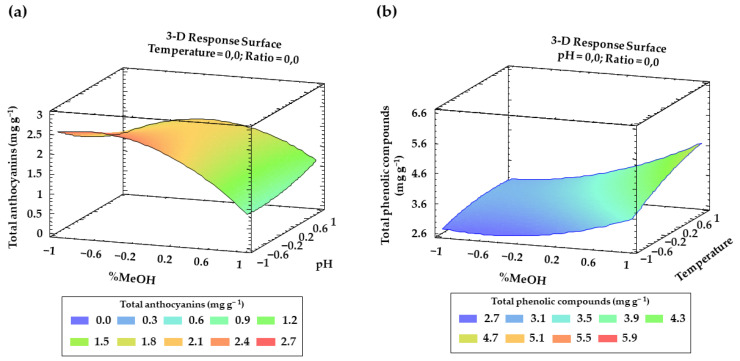
Graphical representation using 3D surfaces of the most significant interactions according to BBD: (**a**) effect of solvent composition and pH on total anthocyanins recoveries (mg g^−1^); (**b**) effect of solvent composition and temperature on the total phenolic compounds recoveries (mg g^−1^).

Finally, for both anthocyanins and total phenolic compounds, from Table 2 and Table 3 and the Pareto charts, a number of interactions between factors and their quadratic interactions with a significant influence on the response can be inferred. These trends have been recorded in three-dimensional (3D) surface plots (Figure 4a,b) using the fitted model for a better understanding.

Based on all the coefficients and effects that have been reported, the polynomial equations that should allow predicting the content of total anthocyanins (Equation (3)) and that of total phenolic compounds (Equation (4)) according to the experimental variables can be determined.

Y_TA_ (mg g^−1^) = 2.03 − 0.51·X_1_ − 0.17·X_2_ − 0.25·X_3_ − 0.03·X_4_ − 0.63·X_1_^2^ + 0.34·X_1_X_2_ − 0.05·X_1_X_3_ − 0.02·X_1_X_4_ + 0.12·X_2_^2^ + 0.03·X_2_X_3_ + 0.09·X_2_X_4_ − 0.21·X_3_^2^ − 0.12·X_3_X_4_ − 0.06·X_4_^2^, (3)

Y_TA_ (mg g^−1^) = 3.28 + 0.63·X_1_ − 0.02·X_2_ + 0.36·X_3_ − 0.21·X_4_ + 0.40·X_1_^2^ + 0.47·X_1_X_2_ + 0.22·X_1_X_3_ − 0.24·X_1_X_4_ + 0.70 X_2_^2^ + 0.70·X_2_X_3_ − 0.17·X_2_X_4_ − 0.16·X_3_^2^ + 0.14·X_3_X_4_ + 0.77·X_4_^2^, (4)

### 3.3. Optimal Conditions

The ANOVA also provided information regarding the values that each factor should take to obtain a maximum response, that is, a maximum anthocyanin extraction on the one hand and maximum total phenolic compounds extraction on the other. Specifically, the following values were established for the optimal extraction of total anthocyanins: 62% methanol in water as a solvent with pH 2, 56 °C extraction temperature, and 0.2:13 g:mL sample-solvent ratio. For total phenolic compounds, the following optimal values were established: 100% pure methanol as a solvent with pH 2, 57 °C extraction temperature, and 0.2:8.8 g:mL sample-solvent ratio. It can be observed, as mentioned above, that the extraction of total phenolic compounds improves as the percentage of methanol is also higher, while the extraction of anthocyanins is better achieved when intermediate percentages of methanol are employed. Regarding pH and temperature, an acidic pH combined with a mild temperature (near the lower value according to the range selected) favor the extraction of both types of compounds.

### 3.4. Extraction Time and Precision

**Figure 5:**
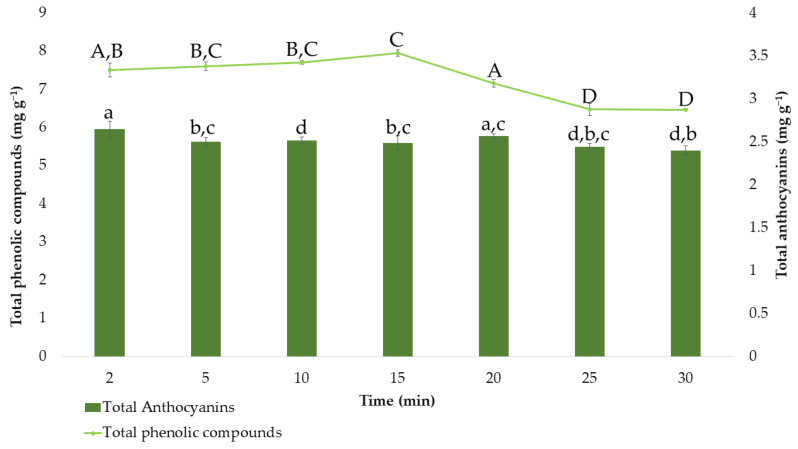
Amount of total phenolic compounds and total anthocyanins (means ± SD) extracted at different times. Different letters mean statistically significant differences according to Tukey’s test at the 95% level of significance.

After determining the effects of the extraction factors on the response variables and the optimal values, the optimum extraction time was also evaluated. Different extractions were carried out under the optimal MAE conditions that had been established so far, while the extraction time varied between 2, 5, 10, 15, 20, 25, and 30 min. The average results obtained (*n* = 3) for total anthocyanins and total phenolic compounds are represented in Figure 5.

Regarding the extraction of total phenolic compounds, it can be observed that in times greater than 20 min, a degradation of the total phenolic compounds occurs. According to Tukey’s test, at 25 and 30 min of extraction (group D), extracts with significant differences and lower amounts of total phenolic compounds are obtained. This is probably because of the degradation experienced by phenolic compounds when the time of extraction is too long [59,62]. Furthermore, the amount of phenolic compounds extracted at 15 min is significantly different from that obtained at any other time (group C). Therefore, 15 min (7.95 ± 0.084 mg g^−1^) was determined as the optimal time since the amount of these bioactive compounds was maximum at this extraction time. Concerning anthocyanins and considering the results of the Tuckey test, there are no significant differences between most of the times studied. Only at a time of 2 min, could it be observed that the amount of extracted anthocyanins differs significantly from the others, only keeping similarity with the amount extracted at 20 min (group a). Consequently, the shortest time, 2 min, which allows time and cost savings with extractions at around 2.64 ± 0.093 mg g^−1^, was established as the optimal extraction time.

Finally, the precision of the two methods when operating under the optimal conditions that had been established has been investigated. For this purpose, a total of 60 experiments were conducted: 30 experiments used the optimal conditions for the extraction of total phenolic compounds, and another 30 experiments used the optimal conditions for the extraction of total anthocyanins. Each set of 30 experiments was carried out on 3 consecutive days by performing 10 experiments each day. In this way, the repeatability of the extraction method could be evaluated as the coefficient of variation (CV) of the experiments carried out on the same day, while its intermediate precision would be determined as the CV of the 30 experiments. Both methods presented good intermediate precision and repeatability, as their CVs were less than 5%, and this is the value generally accepted for this type of assessment [63]. The percentages obtained from the repeatability tests were 2.51% and 3.12%, and the intermediate precision percentages were 3.62% and 4.56% for total anthocyanins and total phenolic compounds, respectively.

### 3.5. Multiresponse-Optimization

In addition to the individual optimization of each of the response variables (total anthocyanins and total phenolic compounds), a multi-response optimization of both extraction processes was carried out. Multi-response optimization is a widely used statistical tool to determine the most optimal conditions to obtain in a single common extraction the greatest possible amount of both total phenolic compounds and total anthocyanins. This is of great interest from the economic point of view since it represents a considerable time and solvent saving. The optimal conditions to equally maximize both responses, total phenolic compounds and total anthocyanins, were the following: 78% MeOH with pH 2, 56 °C, and 0.2:10 g:mL as a ratio. Using these combined conditions and an intermediate extraction time of 5 min, the results obtained were the following: 2.33 ± 0.017 mg g^−1^ for total anthocyanins and 7.05 ± 0.14 mg g^−1^ for total phenolic compounds. These results differ from those obtained from each individual extraction process under their own specific optimal conditions (a *p*-value of the *F*-test less than 0.05). Establishing multi-response optimal extraction conditions seems to be difficult because of the different polarity of the two types of compounds to be extracted. Such polarity differences were evident in view of the dissimilarity between the optimal percentages of methanol in the extraction solvents for the extraction of total phenolic compounds (at 100%) and for total anthocyanins (at 62%). Furthermore, it should also be noted that each specific extraction process has a rather disparate optimal extraction time, with 15 min for the extraction of total phenolic compounds and 2 min for anthocyanins, which poses an additional difficulty when it comes to establishing a single extraction time for both types of compounds. Nevertheless, a combined method would be an interesting alternative for those cases where cutting down time and expenses are essential, such as in quality control laboratories [64]. Furthermore, despite the poorer results, the multi-response method that has been developed in this study achieves greater anthocyanins recoveries than the only other method that has been reported in the literature for the extraction of bioactive compounds from onion using MAE (1.75 ± 0.04 mg C3G/g DW) [29].

### 3.6. Microwave-Assisted Extraction vs. Other Assisted Extraction

**Figure 6:**
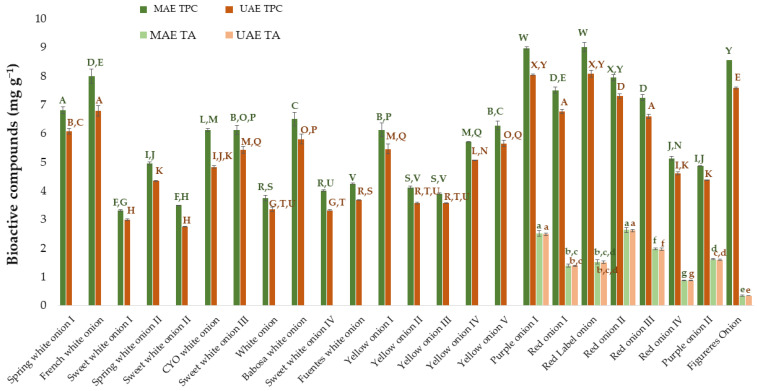
Amount of total phenolic compounds and total anthocyanins extracted by MAE and UAE from different onion varieties. MAE TPC: total phenolic compounds extracted by the optimized MAE method. MAE TA: total anthocyanins extracted by the optimized MAE method. UAE TPC: total phenolic compounds extracted by the optimized UAE method. UAE TA: total anthocyanins extracted by the optimized UAE method. The data in the graph are the mean of 3 replicates ± the standard deviation (expressed as error bars). Different letters mean statistically significant differences according to Tukey’s test at the 95% level of significance.

Finally, to complete this research, the results obtained from MAE were compared against those obtained in a previous work where UAE had been used [25]. For this purpose, the same number and varieties of onion samples subjected to the respective optimal conditions developed for each of the extraction methods were investigated. The results obtained by UAE have been extracted from the previously mentioned work [25]. The TPC and TA obtained from the different onion varieties using UAE and MAE have been included in Figure 6. The ANOVA has been done on the one hand for the results of total phenolic compounds and on the other hand for the results of total anthocyanins. Specifically, the Tuckey test has been applied to evaluate if there are significant differences between varieties and between the two applied extraction techniques (UAE and MAE).

As a result of the Tukey test, it can be observed that there are significant differences (*p*-value < 0.05) between the two techniques for the total phenolic compounds in all the varieties studied. Specifically, it can be concluded that microwave-assisted extraction provides greater amounts of TPC than ultrasound-assisted extraction. Regarding total anthocyanins, although numerically more amount of anthocyanins is extracted using MAE than UAE, statistically and according to the Tukey test, there were no significant differences between the means obtained with both techniques. Regarding the extraction time, as one of the key parameters to be compared and taken into account when it comes to extraction methods, it was equally short for both methods regarding the optimal extraction of anthocyanins, with just 2 min being enough to obtain the best possible results. With regard to the optimal time to extract the total phenolic compounds, MAE took slightly longer than UAE, with 15 min and 10 min, respectively. However, when the same extraction times were compared (5 min for example), MAE provided better results than UAE, with 7.60 ± 0.084 mg g^−1^ extracted by MAE and 6.9 ± 0.14 mg g^−1^ by UAE in the case of the “Red onion II” employed to optimize the processes.

Finally, it is interesting to also analyze the solvent that has been established as optimal for each of the methods since it provides information on the nature of the compounds that were being extracted. The optimal percentages of methanol for MAE were much higher than those for UAE (100% for phenolic compounds and 62% for anthocyanins in MAE versus 53% for phenolic compounds and 57% for anthocyanins in UAE). This indicates that MAE is more appropriate for extracting minor polar compounds, which is why it is more efficient when solvents with a higher percentage of methanol than that of water are used. Considering the “Red onion II” samples used for the process optimization and an intermediate extraction time of 5 min, it can be observed that the main difference concerning the individual anthocyanins lies with anthocyanin 8 (peonidin 3-*O*-(6″-malonylglucoside)), and anthocyanin 9 (delphinidin 3-*O*-glucoside). Thus, greater amounts of both these anthocyanins were extracted by MAE (0.23 ± 0.01 mg g^−1^ of anthocyanin 8 and 0.07 ± 0.01 mg g^−1^ of anthocyanin 9) than by UAE (0.02 ± 0.00 mg g^−1^ for anthocyanin 8 and 0.047 ± 0.00 mg g^−1^ for anthocyanin 9), while the rest of the individual anthocyanins were extracted in similar amounts by both methods. The reverse-phase liquid chromatography that has been applied in this study evidence that anthocyanins 8 and anthocyanins 9 are the less polar compounds present in onion (the last ones to leave the chromatography column, with the longer retention times). So, they are the best compounds to be extracted with a method such as MAE, which has a less polar solvent as previously mentioned.

The developed method has also been compared with other extraction techniques. In regards to assisted extraction techniques, few articles were found: Aguiar et al. [1] reported the use of QuEChERS assisted by ultrasound for the extraction of polyphenols, Stoica et al. [29] and Krithika et al. [65] employed the microwave-assisted extraction, and Hendrawan et al. [66] used ultrasound-assisted extraction. The method of QuEChERS UAE recovered 1.33 mg GAE g^−1^ of total phenolic compounds and 5.13 ± 0.36 µg g^−1^ of total anthocyanins with an extraction time of 5 min and a ratio sample:solvent of 10:10 g:mL, employing pure methanol as solvent. The methods of Stoica et al. and Krithika et al. recovered 1.60 ± 0.05 mg C3G g^−1^ DW of total phenolic compounds and a yield of total anthocyanin content of 21.99, respectively. Regarding the conditions, Stoica et al. used 50% ethanol acidified with 99.5% citric acid at 735 W microwave power for 15 s, and Krithika et al. used 2 g of onion peel with 75 mL of ethanol pure at 700 W microwave power for 5 min. Finally, Hendrawan et al. recovered 1.68 mg GAE g^−1^ of total phenolic compounds employing 20 g of onion with 300 mL of water at 35% of ultrasound amplitude for 5 min. It can be concluded that the times of all the assisted extraction methods are short and of the order of those developed in this work. However, the ratio, and therefore the consumption of samples and solvents, is higher in most cases. Regarding the results obtained, although these vary according to the variety of onions studied in each case, they seem to be of the same order or even lower than those obtained in this work.

Therefore, it can be concluded that MAE is a useful technique for obtaining onion extracts that will allow, among other things, to characterize onion varieties according to their profile of total phenolic compounds and anthocyanins. These extracts with a high content of bioactive compounds will also naturally exhibit a high antioxidant activity that can be of particular interest to consumers. Traditional techniques, such as maceration or sonication, are not really efficient methods to obtain extracts with a high content of bioactive compounds, and, consequently, it is not easy to determine the antioxidant activity of such extracts. In this study, the antioxidant activity of the extracts from all the onion varieties that have been mentioned above has been determined using the DPPH method. The onion extracts were obtained employing the multi-response method which allows obtaining in a single common extraction the greatest possible amount of both total phenolic compounds and total anthocyanins. The results obtained are reported in Table 4.

Based on the results obtained, it can be concluded that the antioxidant capacity of the extracts obtained by MAE is somewhat higher than that of the extracts obtained using UAE [25]. This is in agreement with expectations since, as previously mentioned, MAE is an extraction technique that achieves a greater recovery of bioactive compounds with antioxidant activity, namely total phenolic compounds and anthocyanins. In addition, and based on the onion varieties that have been analyzed in this article, the red varieties generally exhibited a higher antioxidant capacity than the white or yellow ones. This is explained by the fact that only the red varieties contain the anthocyanins that are largely responsible for their red/purple color in their matrix, as can be seen in Figure 6. These coloring compounds contribute to an increased amount of bioactive compounds with antioxidant activity in these onion varieties, and consequently, they exhibit a greater antioxidant capacity [67]. Nevertheless, it should be noted that this reasoning cannot be applied to all the onion varieties studied. According to Tukey’s test, some red varieties do not differ significantly from white or yellow ones. This may be because not only is the color responsible for their antioxidant capacity, but the characteristics of each specific variety also still play a significant role. For example, onion 24, Figueres onion, shows an antioxidant activity that does not differ significantly from a white variety such as onion 10 (group h). The Figueres onion exhibits a purple outer skin, but its inside is rather pale, which would explain its lower antioxidant capacity among the red varieties studied.

## 4. Conclusions

In the present study, two specific microwave-assisted extraction methods have been developed and optimized for the extraction of total anthocyanins and total phenolic compounds from red onions. A Box-Behnken design, together with response surface methodology, was employed to optimize the variables which affect the extraction of the compounds of interest. The variables that have been optimized are as follows: methanol: H_2_O percentages in the extraction solvent, pH of the extraction solvent, extraction temperature, and ratio between the sample weight and the volume of solvent employed. The optimal values for the extraction of total anthocyanins were established as follows: 62% methanol in water as a solvent with pH 2, 56 °C extraction temperature, and 0.2:13 g:mL sample-solvent ratio. Regarding total phenolic compounds, the following optimal values were established: 100% pure methanol as a solvent with pH 2, 57 °C extraction temperature, and 0.2:8.8 g:mL sample-solvent ratio. Both methods exhibited short extraction times (2 and 15 min, for total anthocyanins and total phenolic compounds, respectively), high repeatability, and intermediate precision levels (2.51% and 3.12%, for total anthocyanins, and 3.62% and 4.56%, for total phenolic compounds) and they were able to extract quantitative amounts of the compounds of interest (2.64 ± 0.093 mg g^−1^ for total anthocyanins and 7.95 ± 0.084 mg g^−1^ for total phenolic compounds). Furthermore, a multi-response method was optimized for the extraction of both types of compounds at the same time, which resulted in somewhat lower but with attractive cost and time reductions. Finally, the two optimized MAE methods were successfully applied to different onion varieties, and the extracts obtained exhibited large contents of total phenolic compounds and total anthocyanins, as well as a good antioxidant activity. These results have been compared against those obtained in a previous research work using ultrasound-assisted extraction and with those of other authors. The results confirmed that microwave-assisted extraction is a quantitative, repeatable, and very promising method for the extraction of phenolic compounds and anthocyanins, which offers similar and even superior results with little solvent expense, time, and costs. So, in future works, these methods could be used for the selection of those onion varieties richer in bioactive compounds with antioxidant activity and, therefore, with better biofunctional characteristics regarding health benefits for consumers.

## Figures and Tables

**Table 1:** Box-Behnken design for total anthocyanins (TA) and total phenolic compounds (TPC). The results correspond to the experimental and the predicted values.

Run	Factors				Responses			
	X_1_	X_2_	X_3_	X_4_	Y_TPC_ (mg g^−1^)		Y_TA_ (mg g^−1^)	
					Experimental	Predicted	Experimental	Predicted
1	0	−1	0	0	3.4833	4.0118	2.0412	2.3208
2	1	−1	0	0	5.7664	5.5038	0.8868	0.8339
3	0	1	0	0	3.1456	3.9761	2.0438	1.9783
4	1	1	0	0	4.7861	4.5235	1.2305	1.1776
5	0	0	−1	−1	3.6146	3.8842	2.0176	1.9154
6	0	0	1	−1	3.7680	4.3269	1.6192	1.6535
7	0	0	−1	1	3.6630	3.1813	2.1397	2.1060
8	0	0	1	1	4.3753	4.1829	1.2535	1.3563
9	0	0	0	0	3.2191	3.2825	1.8125	2.0290
10	−1	0	0	−1	4.1747	3.7766	1.8150	1.8508
11	1	0	0	−1	5.3465	5.5262	0.8095	0.8702
12	−1	0	0	1	3.7587	3.8429	1.7955	1.8381
13	1	0	0	1	3.9511	4.6131	0.7089	0.7764
14	0	−1	−1	0	3.5470	4.1913	2.4131	2.3890
15	0	1	−1	0	2.8873	2.7622	1.7712	1.9972
16	0	−1	1	0	3.4709	3.5201	2.0102	1.8339
17	0	1	1	0	5.5978	4.8777	1.4669	1.5407
18	0	0	0	0	3.4444	3.2825	2.3189	2.0290
19	0	−1	0	−1	5.4355	4.8213	2.3473	2.3718
20	0	1	0	−1	5.1206	5.1248	1.9102	1.8571
21	0	−1	0	1	5.0823	4.7371	2.1971	2.1463
22	0	1	0	1	4.0890	4.3621	2.1043	1.9760
23	−1	0	−1	0	2.7386	2.7427	1.9456	1.8986
24	1	0	−1	0	3.8788	3.5676	0.9988	0.9798
25	−1	0	1	0	2.7200	3.0298	1.5266	1.4952
26	1	0	1	0	4.7302	4.7248	0.3750	0.3717
27	0	0	0	0	3.8636	3.2825	2.0626	2.0290

**Table 2:** ANOVA of the total anthocyanins in the MAE extracts.

Source	Coefficient	Sum of Squares	df	Mean Square	*F*-Value	*p*-Value
Model	2.03	7.44	14	0.53	17.29	<0.0001
X_1_: %MeOH	−0.51	2.37	1	2.37	77.05	<0.0001
X_2_: pH	−0.17	0.29	1	0.29	9.54	0.01
X_3_: Temperature	−0.25	0.77	1	0.77	24.96	0.00
X_4_: Ratio	−0.03	0.01	1	0.01	0.28	0.61
X_1_^2^	−0.63	1.94	1	1.94	62.97	<0.0001
X_1_X_2_	0.34	0.19	1	0.19	6.38	0.03
X_1_X_3_	−0.05	0.01	1	0.01	0.34	0.57
X_1_X_4_	−0.02	0.00	1	0.02	0.05	0.82
X_2_^2^	0.12	0.07	1	0.07	2.23	0.16
X_2_X_3_	0.03	0.00	1	0.00	0.08	0.78
X_2_X_4_	0.09	0.03	1	0.03	0.96	0.35
X_3_^2^	−0.21	0.25	1	0.25	8.15	0.02
X_3_X_4_	−0.12	0.06	1	0.06	1.93	0.19
X_4_^2^	−0.06	0.02	1	0.02	0.71	0.42
Residual		0.37	12	0.03		
Lack of fit		0.24	10	0.02	0.38	
Pure Error		0.13	2	0.06		
Cor Total		7.81	26			

**Table 3:** ANOVA of the total phenolic compounds in the MAE extracts.

Source	Coefficient	Sum of Squares	df	Mean Square	*F*-Value	*p*-Value
Model	3.28	16.00	14	1.14	3.06	0.03
X_1_: %MeOH	0.63	3.61	1	3.61	9.66	0.01
X_2_: pH	−0.02	0.00	1	0.00	0.01	0.93
X_3_: Temperature	0.36	1.56	1	1.56	4.19	0.06
X_4_: Ratio	−0.21	0.54	1	0.54	1.44	0.25
X_1_^2^	0.40	0.73	1	0.73	1.96	0.19
X_1_X_2_	−0.47	0.37	1	0.37	0.99	0.34
X_1_X_3_	0.22	0.19	1	0.19	0.51	0.49
X_1_X_4_	−0.24	0.24	1	0.24	0.64	0.44
X_2_^2^	0.70	2.39	1	2.39	6.39	0.03
X_2_X_3_	0.70	1.94	1	1.94	5.20	0.04
X_2_X_4_	−0.17	0.12	1	0.12	0.31	0.59
X_3_^2^	−0.16	0.14	1	0.14	0.37	0.55
X_3_X_4_	0.14	0.08	1	0.08	0.21	0.66
X_4_^2^	0.77	3.37	1	3.37	9.01	0.01
Residual		4.48	12	0.37		
Lack of fit		4.27	10	0.43	3.99	0.22
Pure Error		0.21	2	0.11		
Cor Total		20.48	26			

**Table 4:** Antioxidant activity (*n* = 3) of the different onion varieties extracts obtained by the multi-response extraction method optimized in this study. Different letters mean statistically significant differences according to Tukey’s test at the 95% level of significance.

mg of Trolox Equivalents g^−1^ of Dry Onion ^1^											
Onion 1 ^2^	Onion 2	Onion 3	Onion 4	Onion 5	Onion 6	Onion 7	Onion 8	Onion 9	Onion 10	Onion 11	Onion 12
5.89 ± 0.23 ^a,b^	5.34 ± 0.27 ^a,c^	4.26 ± 0.10 ^d^	4.10 ± 1.34 ^d^	4.87 ± 0.28 ^c^	8.95 ± 0.22 ^e^	6.21 ± 0.49 ^b,f^	8.12 ± 0.18 ^g^	8.25 ± 0.14 ^g^	3.12 ± 0.66 ^h^	4.95 ± 0.59 ^c^	6.72 ± 0.11 ^f,i^
Onion 13	Onion 14	Onion 15	Onion 16	Onion 17	Onion 18	Onion 19	Onion 20	Onion 21	Onion 22	Onion 23	Onion 24
7.89 ± 0.12 ^g^	7.12 ± 0.18 ^i^	4.95 ± 0.29 ^c^	3.89 ± 0.56 ^d^	7.89 ± 0.29 ^g^	6.12 ± 0.02 ^b^	7.21 ± 0.21 ^i^	6.75 ± 0.19 ^f,i^	9.12 ± 0.21 ^e^	9.21 ± 0.12 ^e^	5.45 ± 0.93 ^c^	2.86 ± 0.11 ^h^

^1^ The results are expressed as the mean of three replicates ± the standard deviation (mg of TE g^−1^ of dry onion). ^2^ Onion 1: spring white onion I; onion 2: French white onion; onion 3: sweet white onion I; onion 4: spring white onion II; onion 5: sweet white onion II; onion 6: CYO white onion; onion 7: sweet white onion III; onion 8: white onion; onion 9: Babosa white onion; onion 10: sweet white onion IV; onion 11: Fuentes white onion; onion 12: yellow onion I; onion 13: yellow onion II; onion 14: yellow onion III; onion 15: yellow onion IV; onion 16: yellow onion V; onion 17: purple onion I; onion 18: red onion I; onion 19: red label onion; onion 20: red onion II; onion 21: red onion III; onion 22: red onion IV; onion 23: purple onion II; onion 24: Figueres onion.

## Data Availability

The data presented in this study are contained within the article.

## Abbreviations and Nomenclatures

ANOVA	Analysis of Variance
BBD	Box-Behnken Design
CV	Coefficient of variation
DOE	Design of Experiments
HPLC	High-Performance Liquid Chromatography
MAE	Microwave-Assisted Extraction
*n*	Number of replicates
RSM	Response Surface Methodology
SD	Standard Deviation
TA	Total Anthocyanins
TE	Trolox Equivalents
TPC	Total Phenolic Compounds
UAE	Ultrasound-Assisted Extraction
UHPLC	Ultra-High-Performance Liquid Chromatography
3D	Three-dimensional
X_1_	Percentage of methanol of the solvent
X_2_	pH of the solvent
X_3_	Extraction temperature
X_4_	Ratio between sample weight and volume of solvent
k	Represents the number of factors
C_0_	Represents the number of central points
Y	Represents the responses
β_0_	Represents the model constant
β_i_	Represents the coefficient for each main effect
β_ij_	Represents the coefficient corresponding to the interactions between factor i and factor j
β_ii_	Represents the coefficient of the quadratic factors that represent the curvature of the surface
X	Represents each one of the factors studied
r	Represents the residual value (random error)
